# Annotations

**Published:** 1891-03-07

**Authors:** 


### Annotations.

Mr. Nelson Hardy and Dr. Macnaughton Jones.

Mr. Nelson Hardy is a general practitioner's friend. Nobody can deny that. Moreover, he is himself a general practitioner. The following passage occurs in a letter signed by him and published in the *British Medical Journal* last week: "At the deputation to the Lord President of the Council, Dr. Macnaughton Jones opposed the Bill" (that is the Midwives' Bill) "on the ground that it was fraught with great danger to the public, and that there was no definition in it of the duties of a midwife; but if we turn to his *Hints to Midwives*, which are furnished with his nurses' midwifery and gynæcological bag, by Maw, Son, and Thompson, we shall find that midwives' duties include, according to him, the diagnosis of pregnancy; examination of the urine; the treatment of costiveness during pregnancy; the treatment of abortion; the treatment of convulsions; the administration of laudanum, bromide, and chloral hydrate; stitching up the perineum if ruptured, and the resuscitation of the newly-born by Sylvester's method. It is no doubt due to my dulness of apprehension that I am unable to see how it will increase the danger of the public if women who are already (if in possession of Dr. Macnaughton Jones's *Hints* and bag) considered capable of performing such medical and surgical duties as these, are required in addition to register their names before calling themselves midwives." When a man is very ambitious to come to the front, or very anxious to make money, one of his chief temptations is to "run with the hare and hunt with the hounds." Now if there is one thing more entirely objectionable than another, and more opposed to the code, both of the man of the world and the dignified physician, it is this determination to be at all hazards on the popular and the winning side. It was quite legitimate and proper that Dr. Macnaughton Jones should write a book and call it "Hints to Midwives." But having taken midwives under his wing, it would have been honourable and self-respecting if he had stuck to them. That, however, would have required a certain amount of courage and manhood, and it might have involved the risk of offending a certain number of general practitioners. Dr. Macnaughton Jones, therefore, incontinently forsakes his friends the midwives, and opposes the Bill for their registration, on the ground, according to Mr. Nelson Hardy, that there is in it no definition of the duties of a midwife. But Dr. Jones himself seems to have a very clear idea of the duties of a midwife, and those duties, according to him, are exceedingly comprehensive. If unregistered and uneducated midwives may properly do half the things mentioned by Dr. Jones, then surely it may be permitted to educate and register midwives to do the one simple thing of attending natural labour only. Medical men, it is to be hoped, have sense enough to distinguish between manly consistency and a self-regarding opportunism. It is much to be desired that they will value Dr. Macnaughton Jones's proceedings at their true worth. It will be a bad day for the medical profession when those who trim their sails to catch every possible breeze of popular favour are selected as its leaders.

A Suggested Snake-Bite and Cholera Cure.

The Municipal Commissioner of Baroda has recently invited the consideration of his suggested method of cure for snake-bite and cholera. That any therapeutical procedure which promises a fair hope of success for either scourge should excite the deepest interest in India is not surprising when we remember that many thousand lives are lost in India alone every year from both cholera and snake-bite, and that our present resources are lamentably unsuccessful in coping with these conditions. Mr. Ardishir reminds us of the tradition held by the native populace that the common weasel makes use of certain vegetable roots or leaves for self-preservation when bitten by venomous snakes. While, however, it is certain that the weasel can be bitten with impunity, he believes, and we think rightly so, that the probability is that the weasel is, in itself, proof against any number of snake-bites. Assuming that the weasel must, therefore, contain in its blood an antidote for snake-bites, which is innocuous to human beings, inasmuch as the weasel is commonly eaten by the natives, he suggests the transfusion of weasels' blood into human beings who have been bitten. We are reminded of the recent reports of the curative effects said to have followed the transfusion of the blood of goats into patients affected with tuberculosis, the goat being supposed to be insusceptible to tubercular disease. How far these reports may be credited we do not know, but we feel very strongly that any such practices are based on erroneous premisses. Granted that the weasel and the goat are insusceptible to the virus of snake venom and the tubercle bacillus respectively, there is every reason to believe that such insusceptibility is due to the tissues not being acted on by the virus rather than due to the presence of any antidote in the blood. It is well known that some of the higher vertebrates can feed with impunity on some of the most poisonous herbs known to man, and it is no more remarkable that this should be the case than that strychnine should act so energetically on the anterior cornua of the grey matter of the spinal cord without affecting the posterior cornua, which grey matters, as far as we can ascertain, are chemically identical. But a very slight difference in their chemical constitution must be held to account for the elective affinity of strychnine for the one and its "paraffinity" for the other; and it is fair to suppose that similar modifications in chemical composition of certain tissues account for the insusceptibility of the weasel to snake venom. We know that cobra and rattlesnake-poison kill by paralysing the respiratory nerve-centres in the medulla, and it is to researches such as those of Dr. Lauder Brunton on the action of strychnine in snake-poisoning that we must look for real advances in its treatment. And the same criticism may be applied to the suggested cholera cure, although Mr. Dinishah Ardishir's suggestion that "it may be that in discovering the particular seat of the death-dealing bacilli, we may get hold of a substance for medico-chemical manipulation, which may eventually form a destroying agent for the cholera virus" is analogous to the employment of tubercle virus for the cure of tuberculosis. When he adds that "it may be that an animate object inoculated with the duly manipulated virus may yield a cholera-proof fluid invaluable to mankind," we must recognise that his views will meet with sympathy and support from many of the most scientific investigators in the medical world, and feel assured that he will not have long to wait for the testing of his theories in the physiological laboratory that he so earnestly desires.

Hospitals for All.

The time does not appear far distant when it will become as common a practice to go to a hospital when ill, as to go to a hotel when travelling. There seems no little probability that as hospitals grow in favour—and at present they are very favourably regarded by the public—an ever-increasing number of persons will seek admission for treatment. This is a factor which has not, as yet, engaged the attention of the Lords' Committee. It has also received far too little notice at the hands of the hospital authorities themselves. Formerly nobody dreamt of going to a hospital unless he was absolutely compelled to do so; nowadays, owing to a variety of causes, all this is reversed, and the difficulty is to keep people out, so as to prevent the exclusion of the deserving poor. A movement is now on foot to provide hospital wards for officers of the army and navy. The *Broad Arrow*, which has made itself the mouthpiece of the commissioned officers, regards the non-provision of these wards as proof that an officer in sickness is practically unprotected by the State. Quarters in barracks are often very draughty, and an officer may lie wholly without aid when he most needs it. The isolated situation of such quarters; their distance from the mess-kitchen; the absence of sanitary and nursing arrangements; the evils of the short service system, which prevent the possibility of securing the services of a faithful attendant, are all dwelt upon and emphasized. No doubt there is much to be said in favour of the view advocated by the *Broad Arrow*, and we should like to see the institution of wards for the accommodation of officers of the Mercantile Marine established in connection with the "Dreadnought" Seamen's Hospital at Greenwich. This, at any rate, would meet a pressing want and do an infinity of good.

